# *Platycodi Radix* Extract Prevents Hepatic Steatosis by Enhancing Bile Acid Synthesis in a High-Fat Diet-Induced Fatty Liver Mouse Model

**DOI:** 10.3390/nu16060893

**Published:** 2024-03-20

**Authors:** Wooyoung Kim, Woon Hee Baek, Sung Ho Yun, Hayoung Lee, Mi Jeong Kim, Sang-Yeop Lee, Gun-Hwa Kim, Seung Il Kim, Hye Gwang Jeong, Edmond Changkyun Park

**Affiliations:** 1 Digital Omics Research Center, Korea Basic Science Institute, Cheongju 28119, Republic of Korea; kwy91@kbsi.re.kr (W.K.); bwooon2@gmail.com (W.H.B.); sungho@kbsi.re.kr (S.H.Y.); biryu32@kbsi.re.kr (H.L.); yopi@kbsi.re.kr (S.-Y.L.); genekgh@kbsi.re.kr (G.-H.K.); ksi@kbsi.re.kr (S.I.K.); 2 Critical Diseases Diagnostics Convergence Research Center, Korea Research Institute of Bioscience and Biotechnology, Daejeon 34141, Republic of Korea; 3 Department of Toxicology, College of Pharmacy, Chungnam National University, Daejeon 34134, Republic of Korea; 4 Department of Bio-Analytic Science, University of Science and Technology (UST), Daejeon 34113, Republic of Korea; 5 Immunotherapy Research Center, Korea Research Institute of Bioscience and Biotechnology, Daejeon 34141, Republic of Korea; tito006@kribb.re.kr

**Keywords:** non-alcoholic fatty liver disease (NAFLD), obesity, *Platycodon grandiflorum*, *Platycodi Radix*, proteomics, cholesterol, bile acid

## Abstract

We aimed to identify the mechanism underlying the preventive effects of non-alcoholic fatty liver disease (NAFLD) through *Platycodi Radix* consumption using liver proteomic and bioinformatic analysis. C57BL/6J mice were categorized into three groups: those receiving a standard chow diet (NCD), those on a high-fat diet (HFD), and those on an HFD supplemented with 5% *Platycodi Radix* extract (PRE). After a 12-week period, PRE-fed mice exhibited a noteworthy prevention of hepatic steatosis. Protein identification and quantification in liver samples were conducted using LC-MS/MS. The identified proteins were analyzed through Ingenuity Pathway Analysis software, revealing a decrease in proteins associated with FXR/RXR activation and a concurrent increase in cholesterol biosynthesis proteins in the PRE-treated mouse liver. Subsequent network analysis predicted enhanced bile acid synthesis from these proteins. Indeed, the quantity of bile acids, which was reduced in HFD conditions, increased in the PRE group, accompanied by an elevation in the expression of synthesis-related proteins. Our findings suggest that the beneficial effects of PRE in preventing hepatic steatosis may be mediated, at least in part, through the modulation of FXR/RXR activation, cholesterol biosynthesis, and bile acid synthesis pathways.

## 1. Introduction

In recent decades, obesity rates have risen globally, making it the most common metabolic disorder. Obesity is caused by genetic factors, low physical activity, unhealthy eating patterns, and environmental influences [1,2,3]. The increase in the prevalence of obesity is tightly linked with several chronic health issues, including non-alcoholic fatty liver disease (NAFLD), type 2 diabetes mellitus (T2DM), insulin resistance, dyslipidemia, atherosclerosis, and cancer [4,5]. Excess accumulation of triglyceride (TG) and cholesterol resulting from obesity can damage the liver, which plays a crucial role in systemic metabolism [6,7]. NAFLD is a common, chronic, progressive liver disease encompassing steatosis, non-alcoholic steatohepatitis, cirrhosis, and hepatocellular carcinoma [8,9]. The first stage of NAFLD, hepatic steatosis, is characterized by excess fat accumulation in the liver resulting from an imbalance balance between lipid accumulation and disposal. Pathways in both the uptake and synthesis, as well as the oxidation and secretion, of fatty acids are therefore key in maintaining intrahepatic lipid homeostasis. These processes are controlled by enzymes, hormones, nuclear receptors, and transcription factors [10,11]. Modulating these factors is therefore seen as an effective approach in the prevention and treatment of NAFLD.

Natural products have a long history of medicinal use, and they are still popularly used for alternative therapies [12,13]. The extracts from natural products are multicomponent and possess characteristics that enable them to target multiple proteins. In light of these characteristics, natural product-derived extracts demonstrate various physiological activities such as anti-inflammatory, antioxidant, and immunomodulatory effects. These inherent attributes make them invaluable in the prevention and treatment of various conditions, ranging from tumors and cardiovascular diseases to metabolic disorders and respiratory illnesses [14,15,16]. Consequently, natural products are emerging as a safer and more effective alternative to pharmaceutical agents [17]. Numerous studies have investigated natural compounds as alternatives to pharmacological agents as modulators of metabolic processes. Specifically, natural substances such as resveratrol, green tea, and turmeric have been a significant focus of studies investigating potential NAFLD treatments [18]. These natural compounds can effectively target pathways in the pathogenesis and maintenance of NAFLD without eliciting the adverse effects associated with pharmacological agents. Therefore, natural compounds have high potential as highly tolerable therapeutics able to modulate complex metabolic pathways.

*Platycodi Radix* (PR) is the root of *Platycodon grandiflorum*, a plant belonging to the Campanulaceae family. It is rich in carbohydrate, protein, lipid, ash, and diverse triterpenoid saponins [19]. PR is well known as a traditional herbal medicine for the prevention and treatment of coughs, sore throats, bronchitis, and bronchial asthma in Asia [20]. According to recent reports, extracts and chemical components isolated from PR exhibit anti-inflammatory [21,22], anti-oxidative [23], anti-cancer [23,24,25], anti-obesity [26,27,28,29,30], anti-diabetes [31,32], and hepato-protective properties [33,34]. Furthermore, PR has significant immunomodulatory activity in cellular and humoral immune responses, enhancing the immunogenicity of virus vaccines in mice [35,36]. Most recently, it has been demonstrated that PR exhibits significant anti-obesity and fatty liver preventive effects in diet-induced obese mice [37,38]. Although many studies have reported on the pharmacological properties of PR, the detailed mechanisms underlying the therapeutic effects of PR extract (PRE) are still poorly understood.

The aim and scope of this study were to elucidate the mechanism of action of PRE in preventing NAFLD. To accomplish this, we administered PRE to mice on a high-fat diet (HFD) and conducted a comparative analysis of protein expression by isolating liver tissues for proteomic analysis. Furthermore, we delineated the mechanism of PRE’s action in preventing and treating NAFLD through bioinformatic analysis. As a result of our analyses, we present insights into the mechanism of action of PRE and its potential as a therapeutic strategy against NAFLD.

## 2. Materials and Methods

### 2.1. Preparation of Platycodi Radix Extract

The dried *Platycodi Radix* was obtained from Omniherb (Daegu, Korea). The roots were extracted with 70% EtOH at 50 °C for 6 h, concentrated under vacuum, and then lyophilized, as previously described [37,38]. The dried extract was stored at −20 °C for use in further experiments.

### 2.2. Animals and Diets

This study followed standard animal protocols and obtained approval from the Animal Ethics Committee of the Korea Basic Science Institute (approval number: KBSI-AEC 1815, approval data: 25 June 2018). Five-week-old, wild-type C57BL/6J mice were used. Mice were purchased from Orient Bio Inc. (Seongnam, Korea). The mice were pair-housed in cages at a constant temperature (24 °C) and with a 12 h light/dark cycle, and stress modulation was regulated using diamond twists (7979C.CS, Envigo, IN, USA). Mice were monitored daily by Wooyoung Kim and Woon Hee Baek, who had received training on animal handling from the Korean Association for Laboratory Animal Science. The mice were fed a normal chow diet (NCD, AIN-93G, D10012G, Saeronbio, Uiwang, Korea) for 1 week after arrival as an acclimation period. At 6 weeks of age, they were randomly divided into three groups (*n* = 10 per group) and fed ad libitum with NCD, HFD (60 kcal% fat, D12492, Saeronbio), and HFD supplemented with 5% (*w*/*w*) PRE (the approximate average dose of PRE was 5.46 mg/g bw/day) for 12 weeks. The concentration of PRE was determined according to previous reports [37,38]. At the end of the experimental period, the mice (*n* = 5, randomly selected) underwent a 12 h fasting period and were anesthetized using ether (673811, Sigma-Aldrich, St. Louis, MO, USA) for sacrifice. Blood was taken from the orbital sinus at the time of sacrifice. White (WAT) and brown adipose tissues (BAT) were isolated and weighed. Liver tissue was removed, rinsed with PBS, weighed, immediately frozen in liquid nitrogen, and stored at −70 °C.

### 2.3. Glucose and Insulin Tolerance Test

At the end of the experimental period, glucose and insulin tolerance tests were carried out, after fasting mice for 12 h, by intraperitoneal injection of 1 g/kg glucose or 0.75 U/kg insulin (Eli Lilly and Company, Indianapolis, IN, USA) dissolved in PBS. The blood glucose concentrations were measured from the tail using One Touch Ultra glucometer (LifeScan, Milpitas, CA, USA). Measurements were taken before and at 15, 30, 60, 90, and 120 min after the injection of glucose or insulin.

### 2.4. Histology

Liver tissue was collected for histological examination. Immediately after sacrifice, livers were carefully excised and fixed in 10% formalin. The fixed tissues were then hydrated using a graded series of ethanol and embedded in paraffin. Paraffin blocks were sectioned into 5 µm thick slices using a microtome. Hematoxylin and eosin (H&E) staining was performed on the paraffin-embedded liver tissue sections to assess overall tissue morphology. Stained sections were observed using an ImageXpress Pico (Molecular Devices, San Jose, CA, USA) instrument.

### 2.5. Serum Biochemistry and Liver Lipids Analysis

Blood was collected from the orbital sinus at the time of sacrifice using a capillary tube. Total insulin (ab277390, Abcam, Cambridge, UK), cholesterol (HDL and LDL/VLDL) (ab65390, Abcam), free fatty acids (ab65341, Abcam), adiponectin (ab108785, Abcam), leptin (ab100718, Abcam), and bile acid (ab239702, Abcam), as well as hepatic concentrations of total triglyceride (Wako Diagnostics, Osaka, Japan) and total bile acids (ab239702, Abcam), were measured using commercial assay kits.

### 2.6. Protein Identification through LC-MS/MS Analysis

For LC-MS/MS analysis, peptides were eluted in a gradient of 0–65% *v*/*v* acetonitrile for 120 min. MS and MS/MS spectra were acquired using the orbitrap VELOS instrument (Thermo Fisher Scientific, Waltham, MA, USA) in data-dependent mode. For protein identification, the genome of the mouse UniProt database was searched for MS/MS spectra using MASCOT (version 2.4, Matrix Science, London, UK). Mass tolerance of the parent and daughter ion was set at 0.8 Da. The carbamidomethylation of cysteine and oxidation of methionine were considered as fixed and variable modifications, respectively. A false discovery rate of 1% was used to filter out low confidence peptides, which was calculated using a target-decoy search strategy. MS/MS analysis was repeated three times for each sample (*n* = 3 per group), with the proteins that were identified twice or more being accepted with high confidence.

### 2.7. Bio-Informatic Analysis

Proteins identified via LC-MS/MS were analyzed using Ingenuity Pathway Analysis (IPA, Qiagen, Hilden, Germany). IPA is a web-based software for bioinformatic analysis that identifies network and pathway interactions between proteins, based on an extensive, manually curated database of published interactions. We uploaded the identified proteins and calculated the log_2_ fold change of expression in NCD versus HFD groups, and HFD versus PRE groups, as well as the associated expression value from the proteomics data, into IPA software (version 107193442).

### 2.8. Western Blot Analysis

Liver samples were lysed in RIPA lysis buffer (89901, Thermo Fisher Scientific) containing protease and phosphatase inhibitors (78440, Thermo Fisher Scientific). After centrifuging the lysates (12,000× *g*, 10 min, 4 °C), the supernatant was collected. Protein concentration was measured using a Bicinchoninic Acid Assay Kit (A55860, Thermo Fisher Scientific). Thirty micrograms of protein were loaded into 10% sodium dodecyl sulfate-polyacrylamide gels and separated by electrophoresis before transfer to a polyvinylidene fluoride membrane (IEVH85R, Millipore, Burlington, MA, USA) and incubation with appropriate antibodies: Cyp7a1 (ab65596, abcam); Cyp8b1 (PA5-37088, Invitrogen, Waltham, MA, USA); Bsep (PA5-78690, Invtrogen); and β-actin (3700S, Cell Signaling Technology, Danvers, MA, USA). All diluted buffers were prepared using Tris-buffered saline containing 5% skim milk and 0.1% Tween20. The resulting bands were detected using enhanced chemiluminescence reagents (34579, Thermo Fisher Scientific), and the immune signals were captured using the ChemiDoc image detector (Bio-Rad, Hercules, CA, USA).

### 2.9. Statistic Analysis

All data were statistically processed using the rstatix package in R (version 4.3.2). All graphs were presented with mean and standard deviation, and the confidence intervals were set with an adjusted *p*-value below 0.05. The between-group significance of ANOVA tests was confirmed using Tukey’s multiple comparisons post hoc tests. Data manipulation was performed using the dplyr (version 1.1.4) package, and graphs were generated using the ggplot2 (version 3.4.4) package.

## 3. Results

### 3.1. Preventive Effects of PRE on Obesity and Fatty Liver in an HFD-Induced NAFLD Mouse Model

**Figure 1:**
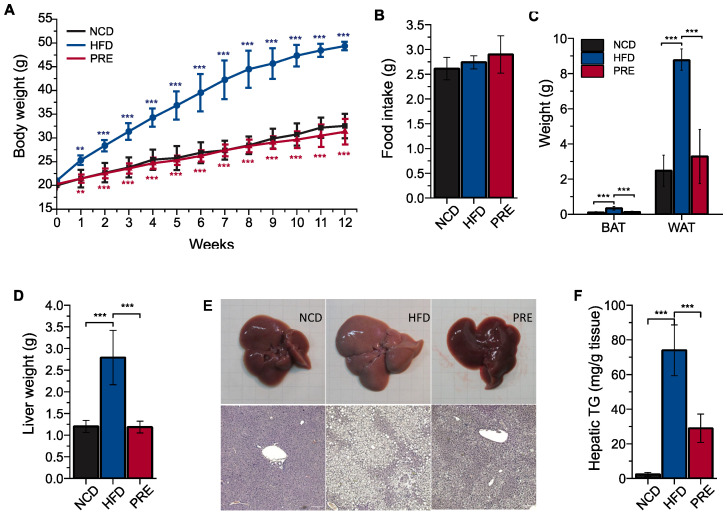
Preventive effects of PRE supplementation on obesity and fatty liver in an HFD-induced NAFLD mouse model. C57BL/6J mice were fed with a normal chow diet (NCD), a high-fat diet (HFD), or a *Platycodi Radix* extract (PRE)-supplemented HFD for 12 weeks. (**A**) Changes in body weight of indicated groups over 12 weeks. (**B**) Food intake. (**C**) Fat mass. (**D**) Liver weight. (**E**) Liver hematoxylin and eosin staining. (**F**) Hepatic triglyceride. Data are shown as means ± SD (*n* = 5 per group). *p* values were determined by ANOVA with Tukey’s multiple comparisons post hoc tests. Statistical differences between two groups are indicated as ** *p* < 0.01, and *** *p* < 0.001. Blue asterisks represent comparisons between NCD and HFD, and red asterisks represent comparisons between HFD and PRE.

To evaluate the potential of PRE in preventing NAFLD, we conducted an animal experiment. The mice were divided into three groups, and each group was fed with NCD, HFD, or HFD supplemented with 5% *w*/*w* PRE. After 12 weeks of diet, HFD-fed mice showed increased body weight compared to NCD-fed mice, but HFD mice treated with PRE (PRE group) exhibited significantly less body weight gain than HFD-fed mice (Figure 1A). However, there was no difference in the amount of food intake among the three groups (Figure 1B). Moreover, PRE mice had significantly lower total fat content and liver mass than HFD mice (Figure 1C,D). These observations suggest that PRE exerts anti-obesity effects in HFD-fed mice. Subsequently, we investigated whether PRE’s anti-obesity effects alleviated hepatic steatosis using H&E staining. As a result, while significant lipid accumulation was observed in the livers of HFD-fed mice, the livers of PRE treated mice showed a reduction in lipid droplets similar to that of normal mice (Figure 1E). This indicates that PRE supplementation effectively inhibited HFD-induced liver steatosis. In addition, PRE significantly lowered triglyceride levels in the liver of HFD mice (Figure 1F). Therefore, our findings suggest that PRE consumption ameliorates the occurrence of obesity-associated NAFLD in HFD-fed mice.

### 3.2. Preventive Effects of PRE on Metabolic Disorders in an HFD-Induced NAFLD Mouse Model

**Figure 2:**
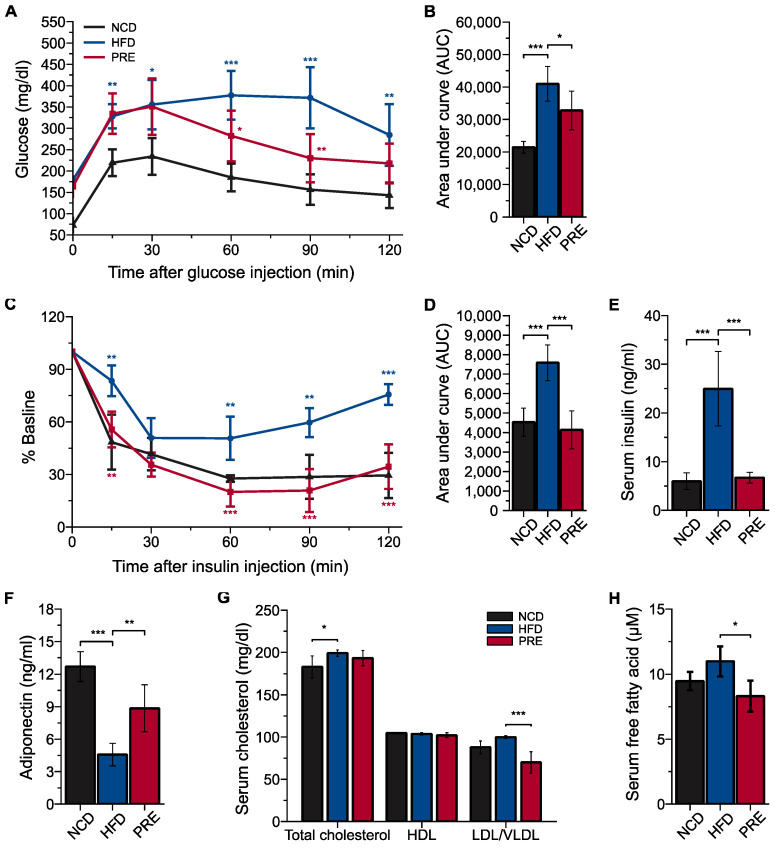
Preventive effects of PRE supplementation on metabolic disorders in an HFD-induced NAFLD mouse model. C57BL/6J mice were fed with a NCD, an HFD, or a *Platycodi Radix* extract (PRE)-supplemented HFD for 12 weeks. (**A**) Glucose tolerance test. (**B**) Area under the curve of glucose tolerance test values. (**C**) Insulin tolerance test. (**D**) Area under the curve of insulin tolerance test values. (**E**) Serum insulin levels. (**F**) Serum adiponectin levels. (**G**) Serum cholesterol levels. (**H**) Serum free fatty acids. Data are shown as means ± SD (*n* = 5 per group). *p* values were determined by ANOVA with Tukey’s multiple comparisons post hoc tests. Statistical differences between two groups are indicated as * *p* < 0.05, ** *p* < 0.01, and *** *p* < 0.001. Blue asterisks represent comparisons between NCD and HFD, and red asterisks represent comparisons between HFD and PRE.

To elucidate whether PRE, beyond its role in mitigating liver steatosis, ameliorated metabolic disorders, we investigated the risk factors associated with both T2DM and hyperlipidemia. The glucose tolerance test showed that PRE recovered impaired glucose metabolism caused by HFD (Figure 2A,B), and the insulin tolerance test demonstrated significantly improved insulin sensitivity with PRE supplementation (Figure 2C,D). Moreover, fasting insulin levels in PRE-treated mice were significantly lower than HFD mice (Figure 2E), while levels of adiponectin, an adipokine secreted by healthy adipose tissue, were significantly higher in PRE mice than HFD mice (Figure 2F). These results suggest that PRE prevents T2DM risk factors in HFD-fed mice. To explore the impact of PRE on hyperlipidemia, we examined serum lipids. There were no significant differences in total cholesterol levels between mice fed different diets; however, LDL cholesterol levels were significantly lower in PRE mice than HFD mice (Figure 2G). Additionally, serum total free fatty acid levels were significantly lower in PRE-treated mice than HFD mice (Figure 2H). These findings suggest that PRE effectively prevents obesity-associated hyperlipidemia in HFD-fed mice.

### 3.3. Proteomic Analysis of the Biological Pathways Underlying PRE Effects in An HFD-Induced NAFLD Mouse Model

**Figure 3:**
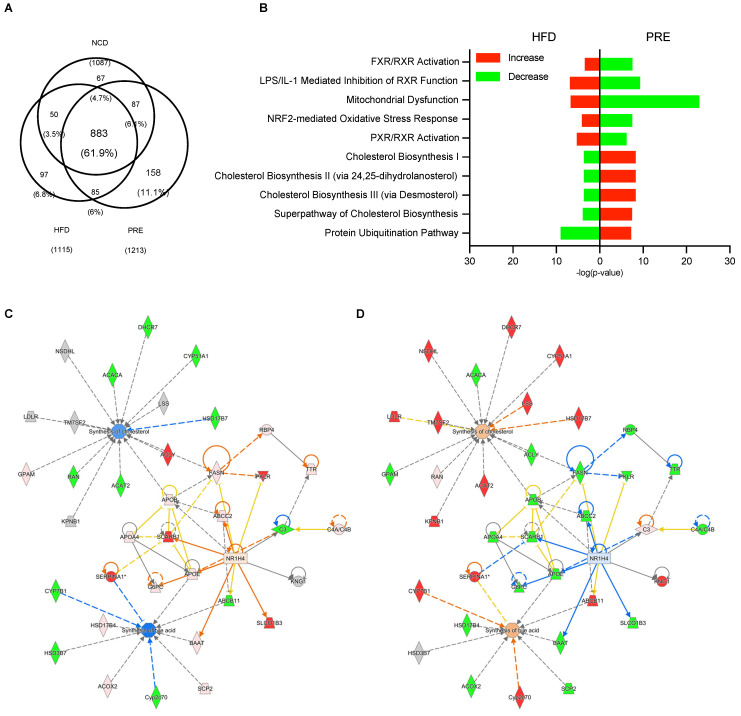
Proteomic analysis of the biological pathways underlying PRE effects in an HFD-induced NAFLD mouse model. (**A**) Venn diagram of identified proteins. (**B**) Canonical pathway analysis. (**C**) Network analysis of the cholesterol biosynthesis pathway in HFD compared with NCD-fed mice. (**D**) Network analysis of the cholesterol biosynthesis pathway in PRE compared with HFD-fed mice. * Red shading indicating increased expression and green shading indicating decreased expression. Orange shading represents active predictions, while blue shading represents inactive predictions. Gray objects represent unidentified genes. The darker the shade of all colors, the higher the value.

Next, to investigate the mechanisms underlying the hepatoprotective effects of PRE in an HFD-induced NAFLD mouse model, we conducted proteomic and bioinformatic analyses. The proteomic analysis identified a total of 1427 proteins, of which, 883 were commonly expressed across all groups. Additionally, 67 proteins were identified in only the NCD group, 97 in the HFD group, and 158 in the PRE group (Figure 3A). Among these proteins, 267 were more than 2-fold higher in the PRE group than the HFD group, while 374 were more than 2-fold lower (Table 1 and Table S1). To explore the impact of PRE treatment on liver proteomics, we conducted a canonical pathway analysis, categorizing proteins that were either higher or lower due to PRE treatment. We identified the top five enriched pathways for both upregulated and downregulated differentially expressed protein (DEP) sets following PRE treatment. These pathways exhibited a pattern opposite to that observed in DEP sets between NCD and HFD groups. Of note, in the DEP set increased by PRE, there was a dense association of pathways related to cholesterol biosynthesis, whereas in the decreased DEP set, there was a close association with the farnesoid/retinoid X receptor (FXR/RXR) activation pathway (Figure 3B).

To further assess the activity of biological functions, we carried out additional network analysis. This analysis was built upon the preceding canonical pathway analysis, with a specific emphasis on closely associated pathways such as FXR/RXR activation and cholesterol biosynthesis. The goal was to minimize biases in expression patterns by leveraging a comprehensive set of DEPs. As a result, PRE was predicted to activate cholesterol biosynthesis and inhibit FXR (Nr1h4). In addition, synthesis of bile acid was predicted to be activated by PRE (Figure 3C,D). These predictions aligned with the established process of bile acid synthesis driven by cholesterol [39,40], as well as current research exploring bile acid dysregulation in NAFLD pathogenesis and therapeutic interventions modulating bile acid signaling pathways [41,42,43]. Thus, the preventive effects of PRE consumption on NAFLD in HFD-fed mice appear to occur through the modulation of bile metabolism in the liver.

### 3.4. Experimental Validation of the Effect of PRE on Cholesterol and Bile Acid Metabolism in an HFD-Induced NAFLD Mouse Model

**Figure 4:**
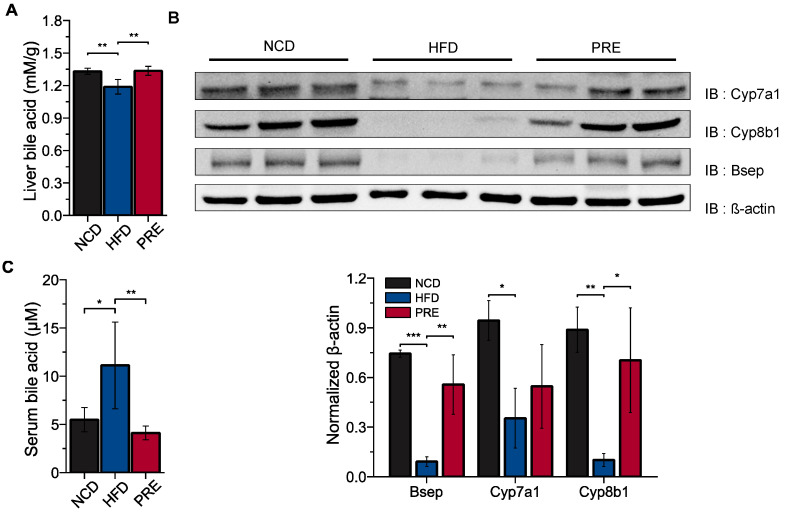
Experimental validation of the effect of PRE in cholesterol and bile acid metabolism in an HFD-induced NAFLD mouse model. (**A**) Liver bile acid level. (**B**) Expression of bile acid synthesis proteins measured by Western blotting in the indicated mouse liver. (**C**) Serum bile acid level. Data are shown as means ± SD. *p* values were determined by the ANOVA with Tukey’s multiple comparisons post hoc tests. Statistical differences between two groups are indicated as * *p* < 0.05, ** *p* < 0.01, and *** *p* < 0.001.

To confirm these predictions, we examined the concentration of total bile acids in mouse livers. In PRE mice, total hepatic bile acids were significantly higher than HFD-fed mice (Figure 4A), which is consistent with predictions from the bioinformatic analysis. To verify the effect of PRE on bile acid metabolism, we further measured the expression levels of proteins using Western blotting. Protein levels of bile acid synthesis enzymes, such as cytochrome P7A1 (Cyp7a1) and cytochrome P8B1 (Cyp8b1), along with the bile transporter bile salt export pump (Bsep), were significantly higher in PRE mice than HFD-fed mice (Figure 4B). These findings suggest that PRE administration alleviates hepatic lipid accumulation in HFD-fed mice by modulating key enzymes and specific transporters involved in bile acid metabolism. In the case of serum bile acids, the amount of circulating bile acids was significantly reduced by PRE treatment (Figure 4C). Collectively, it may be suggested that the preventive effect of PRE on NAFLD is closely associated with the inhibition of bile acid absorption and effects on the distinct pathway of bile metabolism in the liver.

## 4. Discussion

In this study, we tried to elucidate the mechanisms by which PRE prevents NAFLD. To this aim, we first validated the preventive effects of PRE on metabolic disorder, including NAFLD. PRE supplementation exhibited excellent effects in preventing obesity, concomitant with the observed reduction in hepatic lipid accumulation under HFD conditions. Consequently, improvements in glucose tolerance and insulin sensitivity were observed. Furthermore, a significant decrease in circulating insulin, LDL cholesterol, and free fatty acids levels was observed, indicating an overall favorable metabolic profile in PRE-treated mice. These findings are consistent with previous reports [37,38], suggesting the effectiveness of PRE consumption in combating obesity and diabetes. One notable point is that despite the restoration of various physiological factors related to metabolic disease, including insulin sensitivity to NCD levels by PRE consumption, only a partial improvement was observed in the glucose tolerance test. This may be considered to be closely associated with changes in liver triglyceride levels, which are partially reduced in the liver of mice treated with PRE.

Secondly, our objective was to analyze the expression changes in liver proteins induced by PRE supplementation. Through proteomic and bioinformatic analyses, we observed that, compared to an HFD, the set of downregulated proteins in the PRE group was enriched in the canonical pathway of FXR/RXR activation. Additionally, the upregulated protein set showed enrichment in pathways related to cholesterol biosynthesis. FXR plays a pivotal and beneficial role in maintaining hepatic triglyceride balance, influencing glucose metabolism, and regulating bile acid homeostasis. It can reduce hepatic triglyceride content and serum triglyceride levels, leading to improvements in insulin resistance and hyperglycemia [44]. Consequently, FXR agonists show potential as therapeutic options for addressing NAFLD, dyslipidemia, and type 2 diabetes [45]. Interestingly, the FXR/RXR activation pathway and the cholesterol biosynthesis pathway are commonly involved in regulating bile acid biosynthesis [46]. Bile acids are derived from cholesterol via enzymes such as Cyp7a1 and Cyp8b1. Bile acids activate FXR, which in turn suppresses bile acid biosynthesis by inhibiting Cyp7a1 and Cyp8b1 [47].

To further investigate, we conducted the network analysis focusing on the pathway of FXR/RXR activation and cholesterol biosynthesis. The bioinformatic analysis predicted that changes in the liver proteome induced by PRE supplementation would enhance bile acid synthesis. Bile acids act as signaling molecules that regulate liver–intestine crosstalk, and the controlled transport of bile acids between the liver and intestine is crucial for maintaining metabolic homeostasis. Bile acids are synthesized in the liver and then secreted into the small intestine via the bile duct. Upon secretion, bile acids serve to emulsify fats in the intestine and are subsequently absorbed along with fats in the small intestine [44,45,46,47]. Therefore, due to its important role in regulating fat absorption, bile acids are considered a target for obesity control. In fact, studies have shown that inhibiting the reabsorption of bile acids in the small intestine exhibits anti-obesity effects and simultaneously regulates hepatic lipid metabolism [43,48,49,50].

Finally, we validated the changes in bile homeostasis following PRE supplementation. Our results showed an increase in the expression of bile acid synthesis enzymes within the liver, accompanied by elevated hepatic bile acid levels. Furthermore, the reduction in serum bile acid levels indicated a suppression of bile acid reabsorption. These findings align with the predicted network analysis in the liver, providing insights into the effects of PRE consumption on bile acid metabolism.

We experimentally validated the predicted outcomes of PRE supplementation using liver proteome network analysis, thereby elucidating the potential functions of PRE under HFD feeding conditions. The regulatory processes of bile acid absorption and synthesis represent a critical mechanism for maintaining fat and cholesterol metabolism within the body. While many studies aiming to treat NAFLD through bile metabolism concentrate on inhibiting bile synthesis in the liver using FXR agonists [51,52], our results show that PRE led to an increase in bile synthesis, a pattern typically observed when bile acid reabsorption from the intestine is inhibited [53,54]. Bile acids, synthesized in the liver from cholesterol, are stored in the gallbladder and later secreted into the small intestine where they facilitate fat absorption before being reabsorbed into the circulation and returned to the liver [55]. Bile acids absorbed by the liver activate FXR, which, through the action of small heterodimer partner, inhibits the activity of bile acid synthesis enzymes [56]. This process regulates overall circulating levels of bile acids, maintaining a balance in their concentration and activity within the body [46,57]. However, if the reabsorption of bile acids from the intestine is inhibited, the liver responds by activating the synthesis pathway of bile acids using cholesterol [58,59]. Therefore, inhibiting bile acid reabsorption is an alternative target to reduce circulating bile acid levels.

In line with this alternative mechanism of controlling bile acid metabolism, our proteomic and bioinformatic analyses revealed that PRE inhibited FXR and activated cholesterol biosynthesis, in agreement with previous studies investigating changes in the liver transcriptome after inhibiting bile reabsorption via the apical sodium-dependent bile acid transporter [43,49,50]. For the first time, our results revealed that PRE regulates the expression of genes involved in bile acid synthesis and secretion, while lower serum bile acid levels with PRE consumption suggest that the increase in bile acid synthesis was accompanied by an increase in excretion. Overall, our findings reveal a novel mechanism by which PRE exerts its protective effects in obesity-related NAFLD by increasing cholesterol and bile acid synthesis and excretion and by decreasing intestinal reabsorption.

## 5. Conclusions

In conclusion, PRE prevents NAFLD and associated metabolic disturbances by inhibiting intestinal bile acid reabsorption, which increases cholesterol and bile acid biosynthesis and bile acid excretion. This study provides the basis for further research into the preventive application of PRE in the context of metabolic liver diseases and highlights its potential as an effective intervention for NAFLD.

## Figures and Tables

**Table 1:** Summary of quantitative analysis proteome from HFD versus PRE mouse liver.

	Log2 (Fold Change)	Number of Proteins
Only PRE		245
	Log ratio ≧ 1	22
	−1 < Log ratio < 1	719
	Log ratio ≦ −1	227
Only HFD		147

## Data Availability

Data shall be made available upon specific request.

## Supplementary Materials

The following supporting information can be downloaded at: https://www.mdpi.com/article/10.3390/nu16060893/s1. Table S1: PRE MS data.

## References

[1] Buschemeyer W.C., Freedland S.J. (2007). Obesity and prostate cancer: Epidemiology and clinical implications. Eur. Urol..

[2] Ogden C.L., Carroll M.D., Curtin L.R., McDowell M.A., Tabak C.J., Flegal K.M. (2006). Prevalence of overweight and obesity in the United States, 1999–2004. JAMA.

[3] Popkin B.M., Kim S., Rusev E.R., Du S., Zizza C. (2006). Measuring the full economic costs of diet, physical activity and obesity-related chronic diseases. Obes. Rev..

[4] Akagiri S., Naito Y., Ichikawa H., Mizushima K., Takagi T., Handa O., Kokura S., Yoshikawa T. (2008). Bofutsushosan, an Oriental Herbal Medicine, Attenuates the Weight Gain of White Adipose Tissue and the Increased Size of Adipocytes Associated with the Increase in Their Expression of Uncoupling Protein 1 in High-Fat Diet-Fed Male KK/Ta mice. J. Clin. Biochem. Nutr..

[5] Liu Z., Patil I.Y., Jiang T., Sancheti H., Walsh J.P., Stiles B.L., Yin F., Cadenas E. (2015). High-fat diet induces hepatic insulin resistance and impairment of synaptic plasticity. PLoS ONE.

[6] Arguello G., Balboa E., Arrese M., Zanlungo S. (2015). Recent insights on the role of cholesterol in non-alcoholic fatty liver disease. Biochim. Biophys. Acta.

[7] Enjoji M., Yasutake K., Kohjima M., Nakamuta M. (2012). Nutrition and nonalcoholic Fatty liver disease: The significance of cholesterol. Int. J. Hepatol..

[8] Polyzos S.A., Kountouras J., Mantzoros C.S. (2019). Obesity and nonalcoholic fatty liver disease: From pathophysiology to therapeutics. Metabolism.

[9] Angulo P. (2002). Nonalcoholic fatty liver disease. N. Engl. J. Med..

[10] Gluchowski N.L., Becuwe M., Walther T.C., Farese R.V. (2017). Lipid droplets and liver disease: From basic biology to clinical implications. Nat. Rev. Gastroenterol. Hepatol..

[11] Bechmann L.P., Hannivoort R.A., Gerken G., Hotamisligil G.S., Trauner M., Canbay A. (2012). The interaction of hepatic lipid and glucose metabolism in liver diseases. J. Hepatol..

[12] Firenzuoli F., Gori L. (2007). Herbal Medicine Today: Clinical and Research Issues. Evid. Based Complement. Altern. Med..

[13] Chan S.M., Ye J.M. (2013). Strategies for the discovery and development of anti-diabetic drugs from the natural products of traditional medicines. J. Pharm. Pharm. Sci..

[14] Sofowora A., Ogunbodede E., Onayade A. (2013). The role and place of medicinal plants in the strategies for disease prevention. Afr. J. Tradit. Complement. Altern. Med..

[15] Wang L., Li S., Yao Y., Yin W., Ye T. (2021). The role of natural products in the prevention and treatment of pulmonary fibrosis: A review. Food Funct..

[16] Villarroel-Vicente C., Gutiérrez-Palomo S., Ferri J., Cortes D., Cabedo N. (2021). Natural products and analogs as preventive agents for metabolic syndrome via peroxisome proliferator-activated receptors: An overview. Eur. J. Med. Chem..

[17] Moreira D.d.L., Teixeira S.S., Monteiro M.H.D., De-Oliveira A.C.A.X., Paumgartten F.J.R. (2014). Traditional use and safety of herbal medicines. Rev. Bras. De Farmacogn..

[18] Perumpail B.J., Li A.A., Iqbal U., Sallam S., Shah N.D., Kwong W., Cholankeril G., Kim D., Ahmed A. (2018). Potential Therapeutic Benefits of Herbs and Supplements in Patients with NAFLD. Diseases.

[19] Nyakudya E., Jeong J.H., Lee N.K., Jeong Y.S. (2014). Platycosides from the Roots of *Platycodon grandiflorum* and Their Health Benefits. Prev. Nutr. Food Sci..

[20] Li Y., Wan Y., Liu P., Zhao J., Lu G., Qi J., Wang Q., Lu X., Wu Y., Liu W. (2015). A humanized neutralizing antibody against MERS-CoV targeting the receptor-binding domain of the spike protein. Cell Res..

[21] Jang K.J., Kim H.K., Han M.H., Oh Y.N., Yoon H.M., Chung Y.H., Kim G., Hwang H., Kim B., Choi Y.H. (2013). Anti-inflammatory effects of saponins derived from the roots of *Platycodon grandiflorus* in lipopolysaccharidestimulated BV2 microglial cells. Int. J. Mol. Med..

[22] Ahn K.S., Noh E.J., Zhao H.L., Jung S.H., Kang S.S., Kim Y.S. (2005). Inhibition of inducible nitric oxide synthase and cyclooxygenase II by *Platycodon grandiflorum* saponins via suppression of nuclear factor-kappaB activation in RAW 264.7 cells. Life Sci..

[23] Lee J.Y., Hwang W.I., Lim S.T. (2004). Antioxidant and anticancer activities of organic extracts from *Platycodon grandiflorum* A. De Candolle roots. J. Ethnopharmacol..

[24] Lee K.J., Choi J.H., Kim H.G., Han E.H., Hwang Y.P., Lee Y.C., Chung Y.C., Jeong H.G. (2008). Protective effect of saponins derived from the roots of *Platycodon grandiflorum* against carbon tetrachloride induced hepatotoxicity in mice. Food Chem. Toxicol..

[25] Shin D.Y., Kim G.Y., Li W., Choi B.T., Kim N.D., Kang H.S., Choi Y.H. (2009). Implication of intracellular ROS formation, caspase-3 activation and Egr-1 induction in platycodon D-induced apoptosis of U937 human leukemia cells. Biomed. Pharmacother..

[26] Han L.K., Kimura Y., Okuda H., Xu B.J., Zheng Y.N. (2000). *Platycodi radix* affects lipid metabolism in mice with high fat diet-induced obesity. J. Nutr..

[27] Kim K.S., Seo E.K., Lee Y.C., Lee T.K., Cho Y.W., Ezaki O., Kim C.H. (2000). Effect of dietary *Platycodon grandiflorum* on the improvement of insulin resistance in obese Zucker rats. J. Nutr. Biochem..

[28] Zhao H.L., Harding S.V., Marinangeli C.P., Kim Y.S., Jones P.J. (2008). Hypocholesterolemic and anti-obesity effects of saponins from *Platycodon grandiflorum* in hamsters fed atherogenic diets. J. Food Sci..

[29] Lee E.J., Kang M., Kim Y.S. (2012). Platycodin D inhibits lipogenesis through AMPKalpha-PPARgamma2 in 3T3-L1 cells and modulates fat accumulation in obese mice. Planta Med..

[30] Xu B.J., Han L.K., Zheng Y.N., Lee J.H., Sung C.K. (2005). In vitro inhibitory effect of triterpenoidal saponins from *Platycodi Radix* on pancreatic lipase. Arch. Pharm. Res..

[31] Ahn Y.M., Kim S.K., Kang J.S., Lee B.C. (2012). *Platycodon grandiflorum* modifies adipokines and the glucose uptake in high-fat diet in mice and L6 muscle cells. J. Pharm. Pharmacol..

[32] Zheng J., He J., Ji B., Li Y., Zhang X. (2007). Antihyperglycemic effects of *Platycodon grandiflorum* (Jacq.) A. DC. extract on streptozotocin-induced diabetic mice. Plant Foods Hum. Nutr..

[33] Lee K.J., You H.J., Park S.J., Kim Y.S., Chung Y.C., Jeong T.C., Jeong H.G. (2001). Hepatoprotective effects of *Platycodon grandiflorum* on acetaminophen-induced liver damage in mice. Cancer Lett..

[34] Kim H.K., Kim D.S., Cho H.Y. (2007). Protective effects of *Platycodi radix* on alcohol-induced fatty liver. Biosci. Biotechnol. Biochem..

[35] Yoon Y.D., Han S.B., Kang J.S., Lee C.W., Park S.K., Lee H.S., Kang J.S., Kim H.M. (2003). Toll-like receptor 4-dependent activation of macrophages by polysaccharide isolated from the radix of *Platycodon grandiflorum*. Int. Immunopharmacol..

[36] Xie Y., Pan H., Sun H., Li D. (2008). A promising balanced Th1 and Th2 directing immunological adjuvant, saponins from the root of *Platycodon grandiflorum*. Vaccine.

[37] Kim Y.J., Choi J.Y., Ryu R., Lee J., Cho S.J., Kwon E.Y., Lee M.K., Liu K.H., Rina Y., Sung M.K. (2016). *Platycodon grandiflorus* Root Extract Attenuates Body Fat Mass, Hepatic Steatosis and Insulin Resistance through the Interplay between the Liver and Adipose Tissue. Nutrients.

[38] Park H.M., Park K.-T., Park E.C., Kim S.I., Choi M.S., Liu K.-H., Lee C.H. (2017). Mass Spectrometry-Based Metabolomic and Lipidomic Analyses of the Effects of Dietary Platycodon grandiflorum on Liver and Serum of Obese Mice under a High-Fat Diet. Nutrients.

[39] Nagahashi M., Yuza K., Hirose Y., Nakajima M., Ramanathan R., Hait N.C., Hylemon P.B., Zhou H., Takabe K., Wakai T. (2016). The roles of bile acids and sphingosine-1-phosphate signaling in the hepatobiliary diseases. J. Lipid Res..

[40] Zhou F., Sun X. (2021). Cholesterol metabolism: A double-edged sword in hepatocellular carcinoma. Front. Cell Dev. Biol..

[41] Zhang X., Deng R. (2018). Dysregulation of bile acids in patients with NAFLD. Nonalcoholic Fatty Liver Disease-An Update.

[42] Fang S., Reilly S.M., Yu E., Osborn O., Lackey D., Yoshihara E., Perino A., Jacinto S., Lukasheva Y. (2015). Intestinal FXR agonism promotes adipose tissue browning and reduces obesity and insulin resistance. Nat. Med..

[43] Ge M.-X., Niu W.-X., Ren J.-F., Cai S.-Y., Yu D.-K., Liu H.-T., Zhang N., Zhang Y.-X., Wang Y.-C., Shao R.-G. (2019). A novel ASBT inhibitor, IMB17-15, repressed nonalcoholic fatty liver disease development in high-fat diet-fed Syrian golden hamsters. Acta Pharmacol. Sin..

[44] Stofan M., Guo G.L. (2020). Bile Acids and FXR: Novel Targets for Liver Diseases. Front. Med..

[45] Jiao Y., Lu Y., Li X.-Y. (2015). Farnesoid X receptor: A master regulator of hepatic triglyceride and glucose homeostasis. Acta Pharmacol. Sin..

[46] Fang S. (2017). Bile Acid Receptor Farnesoid X Receptor: A Novel Therapeutic Target for Metabolic Diseases. J. Lipid Atheroscler..

[47] Chiang J.Y.L. (2009). Bile acids: Regulation of synthesis. J. Lipid Res..

[48] van de Peppel I.P., Rao A., Dommerholt M.B., Bongiovanni L., Thomas R., de Bruin A., Karpen S.J., Dawson P.A., Verkade H.J., Jonker J.W. (2020). The Beneficial Effects of Apical Sodium-Dependent Bile Acid Transporter Inactivation Depend on Dietary Fat Composition. Mol. Nutr. Food Res..

[49] Rao A., Kosters A., Mells J.E., Zhang W., Setchell K.D.R., Amanso A.M., Wynn G.M., Xu T., Keller B.T., Yin H. (2016). Inhibition of ileal bile acid uptake protects against nonalcoholic fatty liver disease in high-fat diet–fed mice. Sci. Transl. Med..

[50] Matye D.J., Wang H., Luo W., Sharp R.R., Chen C., Gu L., Jones K.L., Ding W.-X., Friedman J.E., Li T. (2021). Combined ASBT inhibitor and FGF15 treatment improves therapeutic efficacy in experimental nonalcoholic steatohepatitis. Cell. Mol. Gastroenterol. Hepatol..

[51] Clifford B.L., Sedgeman L.R., Williams K.J., Morand P., Cheng A., Jarrett K.E., Chan A.P., Brearley-Sholto M.C., Wahlström A., Ashby J.W. (2021). FXR activation protects against NAFLD via bile-acid-dependent reductions in lipid absorption. Cell Metab..

[52] Carr R.M., Reid A.E. (2015). FXR Agonists as Therapeutic Agents for Non-alcoholic Fatty Liver Disease. Curr. Atheroscler. Rep..

[53] Peppel I.P.v.d., Verkade H.J., Jonker J.W. (2020). Metabolic consequences of ileal interruption of the enterohepatic circulation of bile acids. Am. J. Physiol.-Gastrointest. Liver Physiol..

[54] Root C., Smith C.D., Sundseth S.S., Pink H.M., Wilson J.G., Lewis M.C. (2002). Ileal bile acid transporter inhibition, CYP7A1 induction, and antilipemic action of 264W94. J. Lipid Res..

[55] Hofmann A.F. (1999). Bile Acids: The Good, the Bad, and the Ugly. Physiology.

[56] Goodwin B., Jones S.A., Price R.R., Watson M.A., McKee D.D., Moore L.B., Galardi C., Wilson J.G., Lewis M.C., Roth M.E. (2000). A Regulatory Cascade of the Nuclear Receptors FXR, SHP-1, and LRH-1 Represses Bile Acid Biosynthesis. Mol. Cell.

[57] Lu T.T., Makishima M., Repa J.J., Schoonjans K., Kerr T.A., Auwerx J., Mangelsdorf D.J. (2000). Molecular basis for feedback regulation of bile acid synthesis by nuclear receptors. Mol. Cell.

[58] Baghdasaryan A., Fuchs C.D., Österreicher C.H., Lemberger U.J., Halilbasic E., Påhlman I., Graffner H., Krones E., Fickert P., Wahlström A. (2016). Inhibition of intestinal bile acid absorption improves cholestatic liver and bile duct injury in a mouse model of sclerosing cholangitis. J. Hepatol..

[59] Matye D.J., Li Y., Chen C., Chao X., Wang H., Ni H., Ding W.-X., Li T. (2021). Gut-restricted apical sodium-dependent bile acid transporter inhibitor attenuates alcohol-induced liver steatosis and injury in mice. Alcohol. Clin. Exp. Res..

